# Early parameter to detect the resolution of pediatric diabetic ketoacidosis

**DOI:** 10.3389/fped.2025.1570859

**Published:** 2025-04-01

**Authors:** Yi-Hsuan Liu, Ya-Ting Su, Jainn-Jim Lin, Oi-Wa Chan, Chen-Wei Yen, En-Pei Lee

**Affiliations:** ^1^ College of Medicine, Chang Gung University, Taoyuan, Taiwan; ^2^ Division of Pediatric Neurology, Chang Gung Children’s Hospital and Chang Gung Memorial Hospital, Taoyuan, Taiwan; ^3^ Division of Pediatric Endocrinology and Genetics, Department of Pediatrics, Chang Gung Memorial Hospital, Taipei, Taiwan; ^4^ Division of Pediatric Critical Care Medicine, Department of Pediatrics, Chang Gung Memorial Hospital at Linko, Taoyuan, Taiwan; ^5^ Division of Nephrology, Department of Pediatrics, Chang Gung Memorial Hospital, Chang Gung University, Taoyuan, Taiwan

**Keywords:** diabetic ketoacidosis, children, hyperchloremia, resolution, anion gap

## Abstract

**Objective:**

The present study aimed to analyze the incidence of hyperchloremia and compare the time to reach DKA resolution with different parameters.

**Methods:**

A chart review of patients diagnosed with DKA and aged <18 years was conducted. DKA was defined as serum glucose ≧200 mg/dl, venous pH (vpH) <7.30, serum bicarbonate <15 mmol/L, and presence of ketonemia, or ketonuria. Electrolytes and blood gases were recorded at 6-h intervals after treatment initiation.

**Results:**

Overall, 84 patients were admitted because of DKA. The initial biomedical parameters in the emergency department were as follows: median glucose, 497 mg/dl; vpH, 7.1; serum HCO_3_, 6.6 mmol/L; anion gap (AG), 24.7 mmol/L; and ketone, 5.7 mmol/L. After treatment, the incidence of hyperchloremia increased progressively from 15.4% at treatment initiation to 80% at 18 h. The median time to resolution defined by AG ≦12 mmol/L was 12 h, which was significantly faster than the recovery of vpH ≧7.3 (median time, 17 h) and HCO_3_ >15 mmol/L (median time, 18 h). Approximately 63 (75%) patients reached the endpoints of AG ≦12 mmol/L prior, 14 (16.6%) patients reached the endpoints of vpH ≧7.3 prior, 7 (8.4%) patients reached the endpoints of HCO_3_ >15 mmol/L prior.

**Conclusions:**

Hyperchloremic metabolic acidosis (HMA) was a common entity in pediatric DKA following treatment. The median time of AG ≦ 12 mmol/L was approximately 12 h and was the parameter that can identify DKA resolution at a faster rate, i.e., approximately 5, and 6 h faster than the normalization of vpH and HCO_3_, respectively. Future studies were warranted to use AG ≦12 mmol/L as the endpoint of DKA treatment and check if the treatment course and incidence of HMA could be reduced.

## Introduction

Diabetic ketoacidosis (DKA) is a potentially critical complication of type 1 diabetes mellitus (T1D) that mainly causes diabetes-related comorbidity and mortality in pediatric T1D. DKA is present at the diagnosis of T1D in 30%–40% of children in the USA (1–3). Furthermore, approximately one-half of these patients with DKA were diagnosed with moderate-to-severe DKA (pH <7.2) (2, 4). A more severe DKA was associated with higher rates of complications, indicating the need for management by an experienced diabetes team best in the pediatric intensive care unit (PICU).

The common therapeutic protocol for DKA included checking blood sugar hourly and serum electrolytes and blood gases every 4–6 h. The laboratory testing for defining DKA resolution included venous pH (vpH), plasma bicarbonate (HCO_3_), anion gap (AG), and ketone bodies [blood beta-hydroxybutyrate (BOHB) and urine acetoacetate]. The common cutoff value of these biomedical parameters for identifying DKA resolution included blood sugar <200 mg/dl, vpH ≧7.3, HCO_3_ >15 mmol/L, AG ≦12 mmol/L, and BOHB ≦1 mmol/L (5, 6).

Volume expansion with isotonic saline (0.9% sodium chloride) to restore the effective circulating volume is the first step of resuscitation for DKA. Hyperchloremic metabolic acidosis (HMA) usually develops after replacement with large amounts of isotonic saline solution. HMA would result in HCO3 loss to maintain electroneutrality. A previous study demonstrated that HMA was noted in nearly 94% of the patients with DKA after 20 h of fluid resuscitation (7). Recently, von Oettingen et al. reported that the single parameter serum HCO_3_ >15 mmol/L can be used to identify DKA resolution (8). However, previous studies have reported that HCO_3_ >15 mmol/L is probably slower than the recovery of vpH ≧7.3 and AG ≦12 mmol/L in patients with DKA N (7–10). Many clinicians may focus on HCO_3_ >15 mmol/L to indicate DKA resolution. However, HCO_3_ recovery may be masked by HMA, which would result in additional fluid replacement and intravenous (IV) insulin therapy. This additional therapy may result in complications such as cerebral edema and increasing the length of PICU stay (11, 12).

Since only a few studies have analyzed the incidence of HMA in pediatric DKA after treatment and the time it takes for different biomedical parameters to reach DKA resolution. This study aimed to analyze the effect of HMA and compare the time to DKA resolution with different parameters.

## Materials and methods

### Patient population and study design

This retrospective study analyzed all pediatric patients diagnosed with DKA and admitted to the PICU of Chang Gung Children's Hospital between January 2016 and December 2020. This study was approved by the institutional review board of Chang Gung Memorial Hospital (No. 202300264B0).

DKA was defined as serum glucose ≧200 mg/dl, vpH <7.30, serum bicarbonate <15 mmol/L, and presence of ketonemia, or ketonuria (1).

This study enrolled all patients diagnosed with DKA and aged <18 years. Patients who had other notable metabolic disorders were excluded. Electrolytes and blood gases were recorded at 6-h intervals after treatment initiation.

### Definition of DKA severity and therapeutic strategy

The severity of DKA was categorized into three groups according to the acid–base status (8): mild (vpH 7.2 ≤7.3), moderate (vpH 7.1 ≤7.2), and severe (vpH <7.1).

AG was defined as sodium—(chloride + bicarbonate) (reference range 12 ± 2 mmol/L), and AG closure was defined as AG ≦12 mmol/L.

Hyperchloremia was defined as the ratio of chloride to sodium (Cl: Na) >0.79 (9).

HMA was defined as hyperchloremia accompanied by non-AG metabolic acidosis (normal AG).

DKA resolution was defined as vpH ≧7.3, AG ≦12 mmol/L, or bicarbonate >15 mmol/L (5, 8). The treatment protocol for DKA in our hospital recommended normal saline administration during the initial resuscitation in the emergency department. Then, a two-bag system, which had been utilized since the 1990s, was administered in our PICU (10). The two-bag system contains two identical amounts of electrolytes (potassium chloride and potassium phosphate) but different concentrations of glucose. The initial insulin infusion rate was 0.1 u/kg/h. By adjusting the rate of the two bags, different rates of dextrose administration can be accomplished (11).

### Outcome measures

The primary outcome was to analyze the incidence of HMA after treatment. The secondary outcome was to analyze the time required for the three important biomedical parameters (vpH, HCO_3_, and AG) to reach the cutoff values of DKA resolution, and identify which biomedical parameters reach the cutoff values first (e.g., vpH ≧7.3, HCO_3_ >15 mmol/L, or AG ≦12 mmol/L).

### Statistical analysis

Data were reported as mean ± standard deviation (SD) or median (interquartile range).

The Kruskal–Wallis test was used to examine differences in continuous variables.

Univariate analyses were performed using the chi-square test, Fisher's exact test, or Mann–Whitney *U* test, as appropriate. A *P*-value of <0.05 was considered statistically significant. The IBM SPSS Statistics for Windows version 20.0 (IBM Corp., Armonk, NY, USA) was used for all statistical analyses.

## Results

### Patient demographics

In total, 84 patients diagnosed with DKA were enrolled in this study (Table 1). The mean age was 11.1 years, with a male-to-female ratio of 0.8. Approximately half of the patients (*n* = 43, 51.2%) had severe DKA. With increasing DKA severity, fluid administration also increased significantly. The median glycated hemoglobin (HbA1c) was 13.9. The initial biomedical parameters in the emergency department were as follows: median glucose, 497 mg/dl; vpH, 7.1; serum HCO_3_, 6.6 mmol/L; AG, 24.7 mmol/L; and ketone, 5.7 mmol/L.

**Table 1:** Characteristics of the patients (*N* = 84).

Variable	Value
Female no. (%)	45 (53.5)
Age, mean (SD), years	11.1 (4.2)
Weight, kg	36.3 (17.4)
PRISM III	8.9 (8.4)
DKA severity no. (%)	
Mild	14 (16.6)
Fluid (24 h), median (IQR), ml/kg	84.2 (52.1, 104.4)
Moderate	27 (32.2)
Fluid (24 h), median (IQR), ml/kg	86.8 (64.2, 102.1)*
Severe	43 (51.2)
Fluid (24 h), median (IQR), ml/kg	92.7 (74.3, 105.1)*^,^**
Initial biomedical parameters in the emergency department	
Glucose, median (IQR), mg/dl	479 (356, 582)
pH, median (IQR)	7.1 (7.004, 7.181)
Serum HCO_3_, median (IQR), mmol/L	6.6 (4.3, 9.7)
Anion gap, median (IQR), mmol/L	24.7 (20.9, 28)
Ketone, median (IQR), mmol/L	5.7 (5, 6.1)
HbA1c, median (IQR), %	13.9 (12.6, 14.9)
C-peptide	0.22 (0.16, 0.32)
vitamin D	9.3 (7.9, 14.3)
ICU stay, median (IQR), h	44 (37.5, 53)

* *p* < 0.05 (24-h fluid compared with mild DKA).

** *p* < 0.05 (24-h fluid compared between moderate and severe DKA).

**Figure 1:**
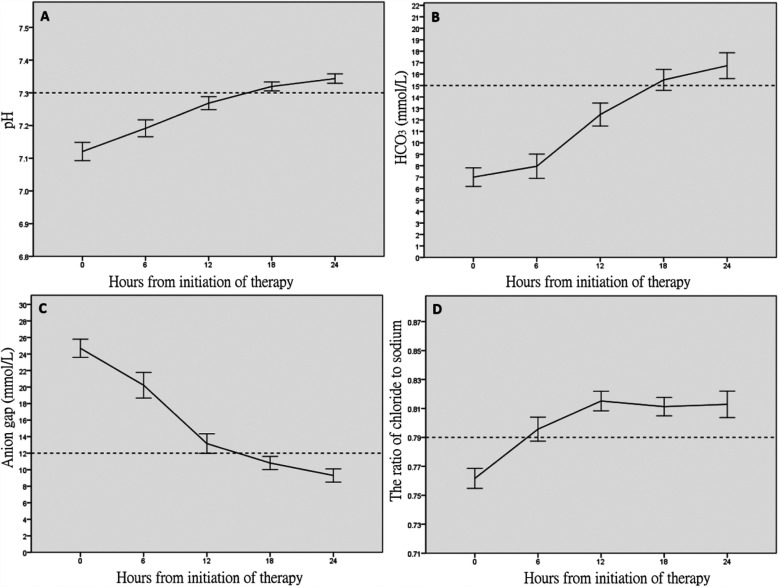
Temporal profiles of the acid–base and electrolyte parameters within 24 h after treatment. **(A)** vpH, **(B)** HCO3 (mmol/L), **(C)** anion gap, and **(D)** ratio of chloride to sodium.

Table 2 shows the temporal profile of the biomedical parameters during the first 24 h of in-hospital treatment. Metabolic acidosis persisted at 18 h (median pH, 7.32; base deficit, 10.7 mmol/L; Figure 1A), although the normalization of bicarbonate (from 6.7 to 15.5 mmol/L, Figure 1B) suggested DKA resolution. High AG persisted for 12 h (from 24.7 to 12 mmol/L, Figure 1C). The incidence of hyperchloremia increased progressively from 15.4% at treatment initiation to 80% at 18 h (Figure 1D).

**Table 2:** Temporal profile of electrolyte parameters.

Time from the commencement of therapy (hours)					
Variable	0	6	12	18	24
vpH, median (IQR)	7.12 (7.03, 7.2)	7.21 (7.15, 7.26)	7.28 (7.23, 7.32)	7.32 (7.28, 7.35)	7.35 (7.32, 7.38)
Serum HCO_3_, median (IQR), mmol/L	6.7 (4.1, 9.3)	6.9 (3.9, 11.2)	13 (9.6, 16)	15.5 (13.6, 17.9)	16.1 (15.1, 19)
Anion gap, median (IQR), (mmol/L)	24.7 (20.9, 28)	17.6 (10.9, 27)	12 (9.5, 16.9)	10.5 (8.6, 12.9)	9.2 (7.3, 10.8)
pCO2(mmHg)	17 (11.4, 22.8)	17.6 (10.9, 27)	28 (22, 31)	30 (27, 33.6)	31 (27.8, 33.8)
Standard base deficit (meq/L)	23.3 (19.6, 26)	21.4 (16.8, 24.3)	13.8 (10, 17.6)	10.7 (7.2, 12.8)	
Sodium, median (IQR), mmol/L	133 (130, 137)	138 (135, 140.5)	138 (136, 141)	138 (136, 141)	138 (137, 140)
Chloride, median (IQR), mmol/L	103 (98, 105)	111 (105.5, 115)	113 (109, 116)	112 (109, 116)	112 (108, 119)
Glucose, median (IQR), mg/dl	479 (356, 582)	234 (183, 284)	189 (139, 226)	180 (133, 225)	172 (143, 215)
Incidence of hyperchloremia (%)	15.4	63	78.5	80	84.5

Values expressed as mean (SEM), pCO2 = partial pressure of carbon dioxide, hyperchloremia was defined as a Cl: Na ratio >0.79.

Table 3 reports the time to DKA resolution. The median time to DKA resolution (defined as vpH ≧7.3 and AG ≦12 mmol/L) was 18 h [interquartile range (IQR) 12–24]. Not unexpectedly, mild DKA (median time to resolution, 12 h) resolved more rapidly than moderate DKA (median time to resolution, 18 h) and severe DKA (median time to resolution, 24 h). The time to resolution defined by AG ≦12 mmol/L was significantly shorter than the resolution time defined by vpH and AG, although shorter than the two discrete endpoints (vpH ≧7.3 or HCO_3_ >15 mmol/L). Approximately 63 (75%) patients reached the endpoints of AG ≦12 mmol/L prior, 14 (16.6%) reached the endpoints of vpH ≧7.3 prior, 7 (8.4%) reached the endpoints of HCO_3_ >15 mmol/L prior.

**Table 3:** Time to DKA resolution (hour).

Variable	All severities (*n* = 84)	Mild DKA (*n* = 14)	Moderate DKA (*n* = 27)	Severe DKA (*n* = 43)
pH ≧7.3 and AG ≦12 mmol/L	18 (12, 24)	12 (8, 18)	18 (12, 18)	24 (18, 24)
HCO_3_ >15 mmol/L	18 (12, 24)**	12 (12, 14)**	14 (6, 18))*^,^**	24 (18, 24)**
pH ≧7.3	17 (12, 24)**	9 (6, 12)*^,^**	12 (6, 18)*	18 (18, 24)*^,^**
AG ≦12 mmol/L	12 (8, 18)*	8 (6, 12)*	12 (8, 16)*	12 (12, 18)*

Data are shown in hours (interquartile range).

* *p* < 0.05 (compared with resolution times, defined as pH ≧7.3 and AG ≦12 mmol/L).

** *p* < 0.05 (compared with resolution times, defined as AG ≦12 mmol/L).

## Discussion

In this study, approximately four-fifths of the patients were diagnosed with moderate-to-severe DKA on the initial presentation. Although the complications were rare in children with DKA, such a high proportion of moderate-to-severe DKA still reminds clinicians that the majority of pediatric patients with DKA need intensive care because these patients need frequent follow-up of biomedical data, more fluid resuscitation, and insulin dosing. Hourly monitoring of vital signs and neurologic symptoms is important in those patients. In addition, the high rate of hyperchloremia increased progressively with time, which indicated that the majority of patients experienced HMA during treatment, which would prolong recovery from DKA. In most of the patients (75%), the AG decreased to normal before the normalization of vpH and serum HCO_3_ levels.

A previous study reported that the development of hyperchloremia during treatment for adult DKA was associated with worsening clinical outcomes such as longer time to DKA resolution, higher rates of acute kidney injury, and longer hospital stay (12). The present study identified a high proportion of hyperchloremia during DKA resuscitation that increased with time, which were comparable with the results of previous pediatric studies (7, 13, 14). Initially, all patients presented with pure keto-acidosis (high AG acidosis), and then 75% experienced secondary HMA (normal AG) during the first 24 h of treatment. The phenomenon was caused by chloride-rich fluids used during treatment, following the renal excretion of anionic ketones (as HCO_3_ precursors), and then chloride was reabsorbed for maintaining electroneutrality (15, 16). The HMA may sometimes mask DKA resolution if the intensivists were not cautious during treatment.

In our cohort, the normalization of AG reflected resolution of DKA faster than vpH and HCO_3_. A previous study reported that HCO_3_ >15 mmol/L maybe a reliable parameter in DKA prediction (8). HCO_3_ recovery was significantly slower than AG closure, and DKA resolution based on the recovery of HCO_3_ and vpH may result in unnecessary treatment. The study identified that AG closure was a reliable parameter for detecting pediatric DKA resolution and occurred 5 and 6 h faster than the normalization of vpH and HCO_3_, respectively.

In clinical practice in the PICU, patients were not transferred to the general ward from the ICU until oral intake and subcutaneous insulin were started; however, this usually did not happen until acidosis had been resolved. HMA developed in the majority of patients with DKA and would prolong the therapeutic course. Previous studies recommended that AG closure can be an early parameter to determine DKA resolution (7, 8), and this study also demonstrated this phenomenon. By using AG ≦12 mmol/L to determine DKA resolution, overtreatment could be avoided (fluid therapy and insulin infusion), oral intake started early, subcutaneous insulin, and ultimately reduction of e the length of PICU stay and medical costs. Future prospective studies are warranted to use AG closure as the endpoint of DKA treatment to determine if it can reduce the incidence of HMA and therapeutic course.

This study is limited by the retrospective design, small sample size, and single-center setting, which could have resulted in information bias. Future studies with a larger number of patients at different centers are needed.

## Conclusion

HMA was a common entity in pediatric DKA after treatment. The median time of AG closure was approximately 12 h and was the parameter that can detect DKA resolution at faster rates, i.e., approximately 5, and 6 h faster than the normalization of vpH and HCO3, respectively. Future studies should use AG ≦12 mmol/L as the endpoint of DKA treatment and determine whether the treatment course and incidence of HMA could be reduced.

## Data Availability

The original contributions presented in the study are included in the article/Supplementary Material, further inquiries can be directed to the corresponding author.
